# Study protocol: multi-parametric magnetic resonance imaging for therapeutic response prediction in rectal cancer

**DOI:** 10.1186/s12885-017-3449-4

**Published:** 2017-07-04

**Authors:** Trang Thanh Pham, Gary Liney, Karen Wong, Robba Rai, Mark Lee, Daniel Moses, Christopher Henderson, Michael Lin, Joo-Shik Shin, Michael Bernard Barton

**Affiliations:** 1 Department of Radiation Oncology, Liverpool Cancer Therapy Centre, Liverpool Hospital, South Western Sydney Local Health District, Sydney, Australia; 2 Sydney West Radiation Oncology Network, Westmead, Blacktown and Nepean Hospitals, Sydney, Australia; 3 Faculty of Medicine, University of New South Wales, Sydney, Australia; 4 Ingham Institute for Applied Medical Research, Sydney, Australia; 5 Department of Radiology, Prince of Wales Hospital, Sydney, Australia; 6 Department of Anatomical Pathology, Liverpool Hospital, Sydney, Australia; 7 School of Medicine, Western Sydney University, Sydney, Australia; 8 Department of Nuclear Medicine, Liverpool Hospital, Sydney, Australia; 9 Faculty of Radiation and Medical Physics, University of Wollongong, Sydney, Australia

**Keywords:** MRI, Rectal cancer, Radiotherapy, Response, Chemoradiotherapy, Diffusion weighted imaging, Dynamic contrast enhanced, Diffusion, Perfusion

## Abstract

**Background:**

Response to neoadjuvant chemoradiotherapy (CRT) of rectal cancer is variable. Accurate imaging for prediction and early assessment of response would enable appropriate stratification of management to reduce treatment morbidity and improve therapeutic outcomes. Use of either diffusion weighted imaging (DWI) or dynamic contrast enhanced (DCE) imaging alone currently lacks sufficient sensitivity and specificity for clinical use to guide individualized treatment in rectal cancer. Multi-parametric MRI and analysis combining DWI and DCE may have potential to improve the accuracy of therapeutic response prediction and assessment.

**Methods:**

This protocol describes a prospective non-interventional single-arm clinical study. Patients with locally advanced rectal cancer undergoing preoperative CRT will prospectively undergo multi-parametric MRI pre-CRT, week 3 CRT, and post-CRT. The protocol consists of DWI using a read-out segmented sequence (RESOLVE), and DCE with pre-contrast T1-weighted (VIBE) scans for T1 calculation, followed by 60 phases at high temporal resolution (TWIST) after gadoversetamide injection. A 3-dimensional voxel-by-voxel technique will be used to produce colour-coded ADC and K^trans^ histograms, and data evaluated in combination using scatter plots. MRI parameters will be correlated with surgical histopathology. Histopathology analysis will be standardized, with chemoradiotherapy response defined according to AJCC 7th Edition Tumour Regression Grade (TRG) criteria. Good response will be defined as TRG 0–1, and poor response will be defined as TRG 2–3.

**Discussion:**

The combination of DWI and DCE can provide information on physiological tumour factors such as cellularity and perfusion that may affect radiotherapy response. If validated, multi-parametric MRI combining DWI and DCE can be used to stratify management in rectal cancer patients. Accurate imaging prediction of patients with a complete response to CRT would enable a ‘watch and wait’ approach, avoiding surgical morbidity in these patients. Consistent and reliable quantitation from standardised protocols is essential in order to establish optimal thresholds of ADC and K^trans^ and permit the role of multi-parametric MRI for early treatment prediction to be properly evaluated.

**Trial registration:**

Australian New Zealand Clinical Trials Registry (ANZCTR) number ACTRN12616001690448 (retrospectively registered 8/12/2016).

## Background

Locally advanced rectal cancer (LARC) requires multi-modality treatment consisting of neoadjuvant chemoradiotherapy (CRT) and standardized surgical technique (total mesorectal excision) [1–3]. Response to neoadjuvant therapy is variable; 15–27% of patients will have a pathologic complete response (pCR) [4], whilst 25–45% will have a poor response with minimal tumour regression [5]. In patients with locally advanced rectal cancer undergoing CRT and surgery, 45% of patients will require permanent colostomy [6]. Accurate imaging for prediction and early assessment of response would enable appropriate stratification of management to reduce treatment morbidity and improve therapeutic outcomes. In patients with a clinical complete response to CRT, substitution of surgery by a ‘watch-and-wait’ approach has emerged as a management option [7–9]. Prediction of poor response could permit trials of dose escalation strategies or curtailment of futile treatment.

Functional magnetic resonance imaging (MRI) has shown promising results for prediction of CRT response in rectal cancer. Diffusion weighted imaging (DWI) has demonstrated greater potential compared with morphologic T2-weighted (T2-w) imaging for the assessment of therapeutic response in rectal cancer patients [10]. However, a systematic review by Joye et al. found that pre-CRT quantitative DWI alone was unable to predict pCR with sensitivity and specificity of 69% and 68%, respectively. Quantitative DWI post-CRT had sensitivity and specificity of 78–80% and 72–78%, respectively, for detecting pCR [11]. Some dynamic contrast enhanced (DCE) MRI studies have shown that higher contrast exchange rate pre-treatment, as indicated by higher K^trans^, is associated with better response to CRT [12, 13]. One study did not find a correlation between pre-treatment K^trans^ and therapeutic response [14]. Use of either DWI or DCE alone currently lacks sufficient accuracy for clinical use to guide individualized treatment in rectal cancer.

Multi-parametric MRI combining DWI and DCE may have potential to improve the accuracy of therapeutic response prediction and assessment. Most published studies describe mean values of a region of interest (ROI) from either DWI or DCE. Single parameter measurements, such as mean apparent diffusion co-efficient (ADC) or K^trans^, do not adequately reflect intra-tumour heterogeneity. A three-dimensional analysis of the tumour volume would provide information on tumour heterogeneity. Development and standardization of multi-parametric imaging protocols is required to provide robust serial imaging datasets and reliable quantitative assessment of treatment response.

### Study hypothesis

Multi-parametric MRI, consisting of DWI and DCE, performed pre-, during- and post- neoadjuvant CRT is predictive of treatment outcome in locally advanced rectal cancer, with histopathology being the standard reference.

## Methods/design

### Study objectives

#### Primary objective

To prospectively evaluate pre-, during- and post-CRT multi-parametric MRI (DWI and DCE) at 3 Tesla for therapeutic response prediction in LARC. MRI biomarkers will be correlated with histopathology tumour regression grade (TRG).

#### Secondary objectives

1. To prospectively evaluate the role of DCE MRI for therapeutic response prediction and assessment in LARC.
2. To prospectively evaluate the role of DWI MRI (RESOLVE) for therapeutic response prediction and assessment in LARC.
3. To evaluate the different contributions of MRI and PET for therapeutic prediction and assessment in LARC.
4. To correlate MRI biomarkers with 2 year disease-free survival and overall survival.

### Study design

The study design is a prospective, single-arm, cohort study to investigate the value of multi-parametric MRI (combining DWI and DCE) in the prediction and assessment of CRT response. Patients will receive standard treatment for their malignancy. This study does not involve a treatment intervention.

### Study schematic

**Fig. 1:**
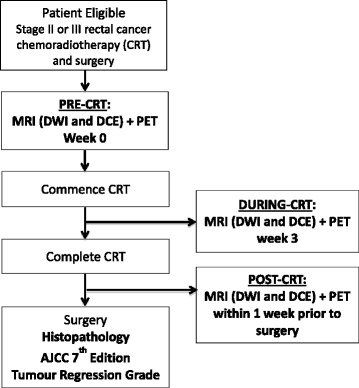
Study schematic

All patients will receive standard treatment consisting of neoadjuvant CRT followed by surgery, and have MRI and PET performed at three time-points (Fig. 1); pre-CRT, during-CRT (week 3 of CRT), and post-CRT (within 1 week prior to surgery).

### Patient selection

#### Inclusion criteria

1. Age greater than 18
2. Stage II or III rectal adenocarcinoma, defined as T3 - T4 and/or node positive disease (N1–2), without distant metastatic disease (M0)
3. No evidence of metastatic disease on computed tomography (CT) chest/abdomen/pelvis
4. Undergoing treatment regimen consisting of neoadjuvant CRT (Radiotherapy 50.4Gy in 28 fractions delivered using 3D–conformal or VMAT technique concurrent with infusional 5-fluorouracil or oral capecitabine) followed by primary surgery.

#### Exclusion criteria

1. Other malignancy
2. Active inflammatory bowel disease
3. Contraindication to MRI:
  ○ Implanted ferromagnetic metal eg. Intraocular metal
  ○ Pacemaker/Implantable defibrillator
  ○ Extreme claustrophobia

#### Treatment

Patients are to undergo standard treatment, consisting of neo-adjuvant long course CRT (as detailed above) followed by surgery, as recommended by treating team. There will be no change to the patient’s treatment from participating in this study.

#### Imaging study procedures

##### Timing of multi-parametric MRI and PET

1. Pre-CRT: week −2 to 0
2. During-CRT: Week 3 of CRT (early as possible during week 3)
3. Post-CRT: Post completion of CRT, within 1 week prior to surgery

### MRI technique

All MRI scans will be acquired on the 3 Tesla Siemens Skyra (Magnetom, Erlangen, Germany) dedicated MRI-Simulator within the Radiation Oncology department. A 32-channel spine coil integrated in the patient table will be used in combination with an 18-channel phase array surface coil strapped firmly around the pelvis. Butylscopolamine (Buscopan) 20 mg will be administered intravenously prior to acquisition of functional sequences (DWI and DCE) to reduce rectal motion.

#### MRI safety screening

All patients will undergo MRI safety screening.

Screening of suitability for gadolinium-based MRI contrast

1. Require documentation of normal renal function within three months (eGFR > = 60 ml/min/1.73 m2
2. Contra-indications to use of gadolinium-based MRI contrast/DCE:
  1. Renal impairment eGFR <60 ml/min/1.73 m2
  2. Acute kidney injury
  3. Previous allergic reaction to gadolinium-based MRI contrast.

#### MRI sequences

##### T2-weighted turbo spin echo images

- Acquired in 3 planes with 2 mm slice thickness and bandwidth 440 Hz/Px in 1 concatenation: (i) sagittal, (ii) axial oblique, angulated perpendicular to the long tumour axis, and (iii) axial. A T2-w HASTE sequence is additionally acquired in the coronal plane.

##### Diffusion weighted imaging (DWI)

- Readout segmented diffusion technique (RESOLVE)
- Axial orthogonal plane with b-values 50 and 800 s/mm^2^, and 1 & 3 signal averages respectively.
- ADC maps and calculated b = 1400 mm/s^2^ images produced as part of protocol.
- In-plane resolution of 1.1 × 1.1 mm and 4 mm slice thickness.

##### Dynamic contrast enhanced (DCE) imaging

- Pre-contrast T1-weighted VIBE scans with TR 4.09 ms, TE 1.35 ms, and flip angles 2° and 15° to calculate native T1.
- DCE acquired with time-resolved angiography with stochastic trajectories (TWIST) technique acquired over 60 phases, with a temporal resolution of 5.28 s, bandwidth 440 Hz/Px, TR 3.67 ms and TE 1.48 ms. Gadoversetamide (Optimark) 0.1 mM/kg injected after 3 phases acquired.

### PET/CT technique

^18^F–FDG PET/CT will be acquired on the GE Discovery-710 PET-CT. Patients need to fast for at least 4 h prior to 4.29 ± 0.34 (mean ± SD) MBq/kg ^18^F -FDG injection and have blood glucose levels <10 mmol/L. All PET scans will be acquired in three-dimensional mode from the mid-brain to proximal femora with an acquisition time of 1.5–2.5 min per bed position, after an ^18^F–FDG uptake time of 60 min. PET data will be reconstructed into a 256 × 256 matrix size with slice thickness of 3.3 mm using GE VUE Point FX (Time of Flight) algorithm. All PET/CT scans will be evaluated on the Advantage Workstation (GE Healthcare) using the AW VolumeShare 5 software and PET-VCAR (Volume Computer-Assisted Reading) protocol.

### Clinical assessment and acute toxicity grading

1. Radiation Oncology clinical assessments and formal acute toxicity scoring will be performed at the following time points:
  - Pre-CRT: Baseline assessment prior to commencement of treatment
  - During-CRT: Weekly during radiotherapy treatment reviews
  - Post-CRT: At 2 weeks post-completion of CRT, and within 1 week prior to surgery (ideally at time of post-CRT MRI).
2. Acute toxicity will be scored according to the ‘Common Terminology Criteria for Adverse Events (CTCAE) Version 4.0.

### Histopathology and tumour regression grade criteria

Histopathological assessment will be undertaken by a dedicated pathology team, with all reporting/TRG assessment by pathologists with sub-specialisation in gastrointestinal pathology. For the study it is a requirement that the whole of the original tumour site should be embedded for microscopic assessment.

MRI will be correlated with histopathological TRG using the modified classification of Ryan et al. [15] set out in the AJCC Cancer Staging Manual, 7th Edition as follows:

*TRG 0 (complete response)* – no viable cancer cells.

*TRG 1 (moderate response)* – single cells or small groups of cancer cells.

*TRG 2 (minimal response)* – residual cancer outgrown by fibrosis.

*TRG 3 (poor response)* – minimal or no tumour kill; extensive residual cancer.

Patients with TRG 0–1 will be categorized as ‘good responders’ and those with TRG 2–3 categorized as ‘poor responders’ to CRT.

### Multi-parametric imaging analysis

A Radiation Oncologist with sub-specialization in gastrointestinal malignancies and a Radiologist with sub-specialization in pelvic MRI will perform all segmentations. K^trans^ maps will be produced by first pre-selecting an appropriate arterial input function, scaled by dose, based on chi-squared goodness of fit, and using a two-compartment Tofts model [16]. ADC and K^trans^ parameter maps will be exported in DICOM format and registered to T2-w axial images.

The region of interest for analysis will be defined on the entire hyper-intense primary tumour on the b-value 1400 mm/s s^2^ images. A voxel-by-voxel technique will be used to produce colour-coded maps and histograms of ADC and K^trans^, and combined scatterplots for each time-point.

### Statistical considerations

A target of 35 patients will be recruited; this is estimated to take 24 months. For estimated pCR rate of 25%, 35 patients will provide 80% power to detect an AUC ≥ 80%. Poor responders rate is estimated to be in the range of 25–45% [5]. For estimated poor responder rate of 25%, 35 patients will provide 80% power to detect an AUC ≥ 80%. For estimated poor responder rate of 45%, 35 patients will provide 80% power to detect an AUC ≥ 76%.

Univariate analysis will be performed to investigate the association between each MRI parameter and tumour regression grade (AJCC 7th edition). Each MRI parameter will be correlated with pathologic complete responders (pCR - TRG 0) using a 2 × 2 Table. A similar analysis will be performed for good responders (TRG0–1) vs. poor responders (TRG 2–3). Receiver operator characteristics curves will be used to obtain an optimal threshold for each MRI parameter (ADC and K^trans^).

Multivariate analysis will be performed to investigate the association between multi-parametric MRI and tumour regression grade. The multi-parametric MRI parameters that will be used for multivariate analysis are ADC and K^trans^.

## Discussion

**Fig. 2:**
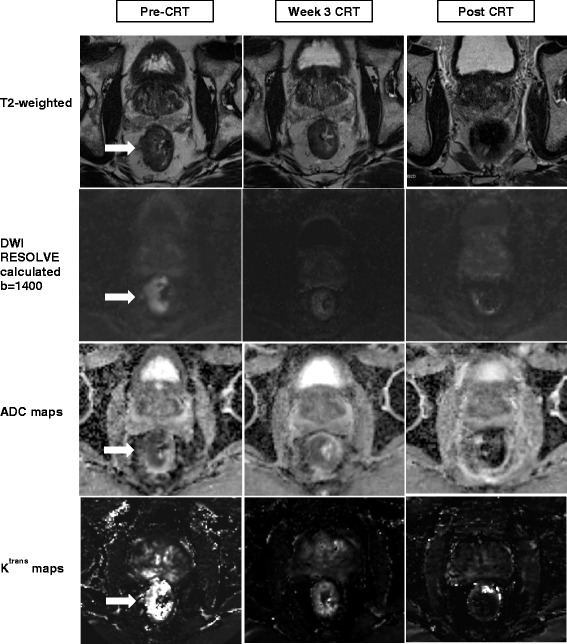
MRI images and functional parameter maps produced as per study protocol. Images for a patient with good response to chemoradiotherapy (CRT) with histopathology tumour regression grade 1 (AJCC 7th Edition). The rectal tumour is indicated by the arrow. In the T2-weighted week 3 image, it was difficult to distinguish residual tumour deposits within areas of radiation-induced necrosis. Early tumour response to therapy can be seen in the functional sequences. In the DWI-RESOLVE images, there was a reduction in tumour signal enhancement in week 3. The calculated b = 1400 mm/s2 images demonstrated increased signal intensity in tumour relative to surrounding normal tissues. Ktrans maps were produced in Siemens Tissue 4D and a reduction in K^trans^ was seen in week 3

MRI offers a variety of functional parameters, with each parameter offering information on various biological aspects of tumour. Multi-parametric image analysis has become increasingly relevant in cancer therapy response predication, as analysis of multiple parameters can provide a more complete physiological assessment of tumour [17]. The rectum is a particularly challenging anatomy to image and provide robust functional datasets that can be examined in a serial manner. In order to provide reliable quantitative assessment of treatment response, it is important to have a standardized protocol that minimizes organ motion and provides robust serial image datasets. This study protocol describes standardized imaging procedures combining DWI and DCE, and a 3D voxel-wise multi-parametric analysis strategy for the assessment of tumour heterogeneity, and prediction of response to CRT in rectal cancer. Both DWI and DCE techniques in this protocol have improvements compared to previously used techniques. The DWI RESOLVE sequence used in this study protocol has previously been shown to be more robust with respect to geometrical distortions, compared to DWI standard echo planar images [18]. The calculated b = 1400 mm/s^2^ images gains from both extra sensitivity and reduced noise of a calculated high b-value. The short temporal resolution of 5.28 s in DCE-TWIST will enable adequate sampling of the rapid wash-in of contrast into tumour. For acquisition of DCE images, the administration of butylscopolamine is crucial in eliminating rectal motion and ensuring accurate signal intensity for each pixel over the 60 time-points. Figure 2 shows examples of the good quality functional images with minimal distortion and corresponding parameter maps that are able to be acquired with this study protocol.

If validated, multi-parametric MRI combining DWI and DCE can be used to stratify management in rectal cancer patients. Accurate imaging prediction of patients with a complete response to CRT would enable a ‘wait and watch’ approach, avoiding surgical morbidity in these patients. Consistent and reliable quantitation from standardized protocols is essential in order to establish optimal thresholds of ADC and K^trans^ and permit the role of multi-parametric MRI for early treatment prediction to be properly evaluated.

## Abbreviations

ADC: Apparent diffusion co-efficient
CRT: Chemoradiotherapy
CT: Computed tomography
DCE: Dynamic contrast enhanced
DWI: Diffusion weighted imaging
LARC: Locally advanced rectal cancer
MRI: Magnetic resonance imaging
pCR: Pathologic complete response
RESOLVE: Read-out segmented sequence
ROI: Region of interest
T2-W: T2-weighted
TRG: Tumour regression grade
TWIST: Time-resolved angiography with stochastic trajectories
